# A Lecture on the Principles and Practice of Dental Surgery

**Published:** 1858-10

**Authors:** Alfred Carpenter


### SELECTED ARTICLES.

### ARTICLE V.

### *A Lecture on the Principles and Practice of Dental Surgery.*

By ALFRED CARPENTER, M. B., M. R. C. S., &c.

[On May the 7th and 14th, 1857, Dr. A. Carpenter gave lectures at the College of Dentists, on the principles and practice of dental surgery. We have been compelled, through the pressure on our space, to postpone the publication of these lectures from time to time, and we fear that we shall be unable to present our readers with the first lecture, which has been unfortunately mislaid by the author, but we have now the pleasure to publish a full report of the second.—*Ed.*]

YOU will recollect, gentlemen, that my first lecture was occupied in detailing the general conditions which give rise to the diseases that affect the different parts of the mouth, or which arise during their course. I described to you, as far as time would admit, the various kinds of inflammation and the various results or events of that action. I mentioned, somewhat cursorily it is true, the various changes that take place in resolution, effusion, adhesion, suppuration, ulceration and mortification. I dwelt somewhat upon hemorrhage, and some of those constitutional causes which occasionally produce mischief in the mouth, and when time compelled me to close my lecture, I had glanced over the action of dentition in the child, and pointed out to you the reasons for and against scarifying the gums. We will, if you please, gentlemen, take up the thread of our discourse at this point; and I will now point out to you some of the diseases proper to the structures of the mouth, and which you will frequently meet with, and be called upon to treat in the course of your practice as dental surgeons. They have names, variously, and at times most improperly, constructed to express their meaning. I will not now enter into a crusade against names, but one cannot help wishing that names used in surgical as well as other branches of science, were framed with the express purpose of meaning something, and not, as is too frequently the case, either meaning nothing, or misleading altogether.

We have considered inflammation generally, let us now look at it locally. It may affect any part of the mouth and throat—the salivary glands, the tonsils, the pharynx, the palate, the uvula, the gums, and the teeth. Thus, the submaxillary glands often become inflamed from cold, or from the irritation produced by the presence of diseased or decayed fangs of teeth in the lower jaw. Recollecting the symptoms of inflammation, and bearing in mind the situation of the sub-maxillary glands, you will easily make out the nature of the case, accompanied, as it always is, by some difficulty in swallowing. Unless you speedily lessen the inflammation, especially if it is the result of diseased teeth, the gland suppurates, discharging very fetid pus, either into the mouth, or externally beneath the jaw. Whenever, therefore, you meet with these swellings below the jaw, accompanied with pain, with heat, and some difficulty of swallowing, do not hesitate to remove every fang of a decayed tooth that may by any possibility be the cause of this inflammation; by this means you may arrest the suppurative process, and save your patient the presence of those unsightly scars which always follow upon processes of this nature. I have at this time under my care, a young lady fourteen years of age, and of considerable personal attractions, irretrievably marked in this manner, which might have been entirely prevented had she consented to the extraction of a decayed tooth at an early period of the disease. The inflammation is sometimes still more extended, and not only injures the superficial structures, but the deeper tissues also; a scale of the alveolar process dies, sloughs, in fact, and has to be exfoliated by a long and tedious process. Little sinuses remain for a long time beneath the jaw, and will not heal by any surgical appliances until the dead portion of bone has been removed. This may not occur for many months, during which, a thin sanious discharge takes place, irritating the surrounding skin as well as the patient's temper, and sadly trying that of her medical attendant. At length the probe detects a loose piece of bone; it is seized by the forceps and quietly extracted; and now the sinus heals in a very short space of time. Now, you will ask, can we not do something to hasten this process? The answer will be—No! If you have removed all dead fangs from that portion of the mouth, and if there is no depot of matter to be felt beneath the gums, you had better let further proceedings alone. You might inject solutions into your sinus to try and hasten the separation, but you are just as likely to increase the size of the part to be separated: therefore, your best plan will be to wait quietly until nature has kindly separated it for you: then your aid will be necessary and useful in removing the little sequestrum of bone with a pair of dressing or bone forceps. Your patient should simply keep a little water dressing upon the wound, and cover it up with oil-silk, whilst every means should be used to improve the general health.

Inflammation of the parotid gland, or the mumps, strongly alters the appearance of the mouth, and, in the early stage, might be mistaken for toothache. I will, however, not stay to consider it, but pass on to inflammation of the inside of the mouth. This, sometimes, puts on an appearance called aphthæ, from the Greek Απτω, to burn. The tongue, the roof of the mouth and the gums, exhibit several little white or grayish spots, which look like ulcers, and are frequently described as such. In the majority of instances, however, this is not the case, they are really little blisters upon the surface of the skin—exudations from an inflamed membrane; and when these exudations are brushed away, the exposed cutis, or more properly speaking, the basement membrane is very red and tender, causing more or less pain when food is taken. These blisters are exceedingly annoying when a few only exist, and a very serious malady if numerous, when a disease is produced called thrush, common in early infancy, as well as during first dentition, and also frequently met with in the latter stages of some exhausting diseases, as consumption, &c. Small ulcers will, however, exist in the mucous membrane of the mouth—the mucous exudation falls off, and the basement membrane actually ulcerates, sores form, varying in size from half a pea to a surface the size of a sixpence, sometimes deep, with thickened edges, and presenting an ashy white color, and when on the side of the tongue, or near the throat, causing some inconvenience and difficulty in swallowing. These ulcers are frequently produced by the sharp edge of a broken tooth; a portion of a carious tooth breaks off and leaves a sharp edge, which first blisters the tongue, and the irritation being continued, an ulcer follows. They may also be caused by the edge of a plate which does not fit the gum evenly, and which irritates the tongue in the same manner, and I have occasionally had reason to believe, that it has been caused by a person's own fault in going to a pretended dentist, who has made use of base metal instead of gold in the formation of the plate itself; by means of which a galvanic action is kept up in the mouth, to the serious detriment of the patient's mucous membrane, as well as the remaining teeth. These ulcers are best remedied by touching them with a stick of nitrate of silver two or three times, and removing the exciting cause, if it is in the mouth; if it is not there, but in the constitution itself, by handing your patient over to the physician, for his general health requires attention.

The gums are very liable to inflammation; you have redness, swelling, heat of mouth, soreness, and often very severe pain. The common cause is irritation, arising from dead fangs or diseased teeth. Now I told you, when speaking of inflammation, that when a part mortifies, nature sets up a process of separation—a line of demarcation is found separating the dead from the living. The ulcerative process gradually eats through all intervening structure, and the dead part falls off if it be not removed by surgical aid. The same law affects the action set up by foreign bodies; if a spiculum of wood be run into the finger, inflammation arises, and after some time, the offending or intruding body is expelled. It is a fundamental law of the animal frame, that dead parts shall irritate living parts, and produce such conditions as naturally lead to the expulsion of the offending agent. But the teeth are so firmly set in their sockets, and are so dense in their structure, that they do not easily ulcerate or absorb; they remain, therefore, for a long time after their complete or partial death, a plague to the soft parts with which they are connected; they keep up a state of chronic inflammation, the gums around become exceedingly sensitive, and are easily excited into acute inflammation by the action of irritants. If the unfortunate possessor be exposed to the action of cold, or the gums be irritated by increased acidity of the secretions of the mouth, or by any exciting cause of inflammation, this acute inflammation is remarkably prone to end in abscess, gum-boils, as the little ones are called; sometimes the quantity of matter formed is very large, and of an exceedingly offensive character.

The chronic form of inflammation is frequently attended by ulceration of the gum around the decayed tooth, the soft parts separating therefrom, in the vain attempt to expel the offending body. This process goes on sometimes for a very long period; fungous granulations spring up, unhealthy, spongy, loose, and incapable of cicatrizing; they easily bleed and make the mouth exceedingly disagreeable, and whilst the tooth remains in situ, no satisfactory cure of this state of things can possibly take place. How vain then must be the promise of the dentist, who tells his patient that he will fit him a set of teeth without extracting the stumps ; and if the dentist knows the impossibility of such being of service, how unwise and injurious to the best interests of the dental profession. You may depend upon it, that whilst there are dead teeth, or ulcerating fangs remaining in the jaws, you will never be able to fix a plate in the mouth that your patient can wear with comfort; if you do enable him to wear it, he will not wish to use it for any length of time.

When the inflammation exists in the gum itself, the matter is generally formed within the substance of the gum, and can be easily evacuated ; but it is frequently far more serious in its nature, the matter forming between the substance of the gum and the alveolar process, or it may even be, within the socket of the tooth. If the suppurative process is established here, the pain is very severe, and may continue without intermission for many days; eventually the matter is discharged, and there is at once a great alleviation of the suffering. It may discharge near the seat of inflammation, or it may burrow along the bone beneath the periosteum, for some distance, and then discharge itself, forming a fistulous opening, through which putrid fluids are continually finding their way into the mouth, to the great inconvenience and disgust of the patient.

This discharge of matter may take place into the antrum, and then the case becomes still more serious ; for the whole of the mucous lining of that cavity may take on the inflammatory process. It occasionally happens, that this effect of previous disease, is the condition attended to most, whilst the original cause of the mischief, viz.—a portion of a diseased tooth is left alone, only to do greater mischief at some future period. When the membrane of the antrum inflames from this cause, it pours forth a vitiated secretion, which is not exactly pus, but a kind of mucus altered in quality, and increased in quantity by inflammatory action. This condition of the antrum can of course only arise when the inflammation of the gums has had its origin in diseased fangs of the upper teeth, and if you recollect the anatomy of this part of the mouth, you will easily see how such an event may arise; for the teeth of the upper jaw frequently send their roots very nearly through the floor of the antrum, and at times they are merely covered by the lining membrane of that cavity.

The pain attending upon inflammation of the antrum, will depend very much upon the pressure exerted by the effused fluid, and the structure of the antrum is such, that it must be filled before the pressure becomes great: the pain then is continuous and urgent, the walls of the cavity become distended, the cheek may project, and at length matter breaks forth through the nose or mouth, and relief is obtained by nature, if art does not come first to aid, by evacuating it through the alveolar processes of those teeth which first set up the mischief.

The best evidence of suppuration within the antrum, in addition to the uneasiness and tenderness of the cheek, will be the flow of matter through the apertures left, when decayed teeth have been removed, or by its discharge into the nose.

The treatment of suppuration within the antrum, consists in the formation and maintenance of an opening in a depending situation, and occasionally syringing it out with warm water. If there is no tooth requiring removal, the antrum may be perforated through its anterior parietes. Divide the mucous membrane and periosteum above the first molar tooth, and perforate the now thinned bony wall: be careful to make sufficient allowance for the depth of the alveolar process, and direct the perforator obliquely upwards and inwards; make your opening large, and prevent its closing, by occasionally inserting a slip of lint, or a small wax tent. You will now have a fistulous aperture which will give you some trouble to close, unless you have removed the cause of the suppuration in the first instance; this will generally be the stumps of decayed teeth, and all the fangs must be removed as soon as they are discovered.

Sir B. Brodie says, in a clinical lecture, reported in the Medical Gazette:—"The cause in which the disease (*i. e.* suppuration of the antrum) originates, is generally a diseased tooth, which, by-the-by, gives rise to toothache. The inflammation on which the toothache depends then terminates, as it always does, in the death of the pulp of the tooth: then the whole tooth dies, and is now like a portion of dead bone, or any foreign substance stuck in the jaw."

The teeth are liable to a peculiar calcareous deposit around their crowns, called tartar, or salivary calculus; it adheres very firmly to them, and insinuates itself under the edges of the gums, exciting irritation, and at length inflammation and ulceration. It is deposited from the saliva, and is generally found in larger quantities near to the ducts of the salivary glands, especially those of the lower jaw. The formation of tartar, its chemical characters, and the reasons for its presence in some parties, and not in others, are at present doubtful points; one author represents it as a secretion from the saliva itself, whilst another states, with some degree of reason, that it is composed of the calcareous shells of innumerable animalculæ, or vibriones, which infest the mouth, leaving their debris to crystallize (so to speak) around the teeth. Berzelius has analyzed it, and states, that it consists of—

| | |
|---|---|
| Phosphate of lime and magnesia | 79.000 |
| Salivary mucus | 13.5 |
| Animal matter | 7.5 |
| | 100.0 |

The mucus is derived from the saliva, and is evidently the bond of union for the calcareous particles.

We now come to consider the treatment of inflammation of the gums. If tartar be present, it must be removed as fast as deposited. If the teeth are well brushed every day, it will not form, but when this process has been neglected, it adheres so firmly to them, that a steel instrument is necessary for this purpose, and you are called upon to scale the teeth.

If the inflammation has arisen from decayed teeth, these must be removed; for so long as a dead fang is left in the jaw, there can be no hope of removing the inflammation.

Sometimes the inflammation is caused by the presence of teeth which are not sound, while, at the same time, they are not sufficiently decayed to justify removal; there may be some little irritating cause near the teeth, keeping up inflammation in the gum, and assisted by the carious tooth, leading to continual danger of suppuration. This should be remedied by lessening the inflammation by local bleeding; a leech skillfully applied to the actually inflamed part, will frequently be of great service—scarifications are sometimes used with success, but the success is more doubtful than leeching; cold water is very useful, carefully and continually applied; it is only, however, completely serviceable in slight inflammations, leeching being by far the most effectual remedy, for the flow of blood will continue for some time from a leech-bite, whilst a scarification ceases to bleed in a few minutes, so that there is no time for the inflammation to become subdued. It is a remedy, however, which must be used in the early stage of the disease, as after suppuration has taken place, it will be of no service; your object should then be to hasten the evacuation of the pus by emollients and fomentations generally; at the same time the general health must be attended to. When you are quite certain of the presence of pus, and if the abscess can be easily reached, a lancet should be passed into it, and afterwards a plentiful supply of warm water used. When the gums are what is called very spongy, scarifications are of most use, and considerable benefit is obtained by using a wash, consisting of gallic acid and tincture of myrrh, once or twice a day. At all other times, applications are worse than useless, the majority are decidedly injurious, and your patient had much better use cold water than any of the many stimulating and acid substances, which are so frequently recommended as washes for the mouth when the gums are inflamed.

A very frequent source of inflammation in the young adult is the irritation set up during the dentition of the wisdom teeth; I shall have a few words to say hereafter, regarding these organs; and I will now only remark, that free scarifications will relieve, assisted by a good dose of physic; for just as in young children, dentition is rendered more easy by a free condition of the alimentary canal; so likewise in adults, the wisdom teeth are always cut with difficulty when the digestive organs are at fault.

I mentioned to you, when I spoke of inflammation, that it might be common, or specific; the common being that inflammation which is excited at any time by any thing, but the specific can only be excited by one agent; thus we may have the rheumatic, the mercurial, and the syphilitic, each of which can only be excited by rheumatism, by mercury, or by syphilis. Now the rheumatic inflammation differs considerably from the others, inasmuch as it is not prone to suppurate; you need not fear the supervention of this event, and your treatment, therefore, will not be directed so much to the local pain, as the general state of the system; your patient wants medical treatment; cold or warm water will be all the local appliances necessary; but it is most likely that a blue-pill and a black draught, followed perhaps by a few doses of quinine, or a glass or two of wine, according to the strength of your patient, will be much more likely to remove the pain than leeching or scarifying the gums.

Mercurial inflammation is another condition, very likely to be frequently brought to your notice, although salivation is not carried to the great extent that used to be the case in days gone by. Now I should tell you, that when mercury has been given for a period of time, varying according to the constitution and temperament of your patient, the gums become spongy and congested; there is an increased flow of the salivary and mucous secretions into the mouth; their color changes to that of a pale rose, with a border of bright red next the teeth themselves. The soreness and swelling increase, the red line near the teeth ulcerates, the discharges become excessive, and a constant coppery taste is present in the mouth; the inflammation increases, the gums blister all over, are excessively sore, irritative fever sets in, the tongue swells, is pushed against the teeth, the saliva flows in quantity, the inflammation increases, and it may be that gangrene results; while the patient grows weaker and weaker, the red blood departs from the body, excessive palor results, and possibly death closes the scene. Such is the progress of events which *may* follow upon a long-continued, or an injudicious course of mercury. The symptoms, however, may cease at any point in the course or train I have mentioned. The most rational plan of treatment is to abate the inflammation by leeches beneath the jaws, used several times in succession, and in the interim applying a large linseed-meal poultice, and using a wash for the mouth, consisting of a weak solution of brandy and water, changing it occasionally for a lotion containing a drachm of solution of chloride of soda, in an ounce of water; this latter has a most beneficial effect in cleansing the intolerable fetor of the breath, and allows the patient to take food with some degree of comfort. This kind of wash will always be the best that can be used, in all cases when the discharges are of an offensive character, and will, by correcting the frequently-disgusting odor of these cases, be a great boon to both surgeon and patient. When there is only a slight amount of salivation, the leeches will of course not be required; I should only recommend their application when the inflammation is of a character to threaten gangrene.

There is another medicine which easily affects the salivary glands and gums—it is iodine and its different salts, especially the iodide of potassium, a medicine of great power and varied use. Whilst a patient is under its influence there is a great increase in the nasal mucus, he is frequently clearing his throat, the gums become spongy and tender, of a darker color than from mercurial salivation, whilst the increase of secretion is more that of mucus, than of the salivary fluids. If there are any decayed or decaying teeth in the mouth, the exhibition of the iodine may be the means of increasing that decay, and exciting actual inflammation around the tooth, leading, it may be, to suppuration; when such is the case, you should advise the suspension of the remedy, unless the disease for which it is given is of more consequence than the maxillary ailment; the increased excitement of the gums and dental nerves will usually subside after a few day's rest from the action of the iodine, whilst all your attention will not avail if the use of the medicine be persevered in. Now neither mercury nor iodine will do positive injury to the teeth, if these are otherwise healthy, and the secretions of the buccal and other salivary glands are healthy in their character. If, however, these latter are unhealthy, that is, if they are changed, and have an acid reaction, instead of an alkaline, which they ought to have; then the influence of both those medicines may act most perniciously.

There is another agent which acts directly upon the gums; one that is occasionally used as a medicine, but much more frequently obtains entrance into the system, when a man is engaged in the fine arts, from being exposed to its fumes, or from its being accidentally present in water—I mean lead and its compounds. I do not know that it has any active action upon the teeth, but I never see the evidence of its presence, unless the teeth are much decayed; and they are always more or less stained brown. The presence of lead in the system is known by a narrow leaden-blue, or slate-blue line, from one-twentieth to one-sixth of an inch in diameter, on the margins of the gums nearest to the incisors of either jaw, the rest of the gums being of a bluish-red tint. This blue discoloration depends upon the presence of sulphuret of lead in the system, and requires general treatment. In no case ought dental operations to be performed upon the mouth, at least operations for the purpose of fixing new teeth, until all evidence of lead discoloration has disappeared, for the gums will be somewhat swollen, and the plate cannot possibly rest comfortably upon a membrane loaded with sulphuret of lead.

I may as well, whilst I am upon the subject of inflammation, tell you a little about the wisdom teeth. Now we are all of us alive to the complications attending the dentition of infants; but the second takes place so gradually, and usually with so much ease, that we are to forget that such is not always the case, that complications may and do arise of a most serious character; inflammation of brain, diarrhea, convulsions, and a series of other maladies, come on and pass away, frequently without any reference being made to the proximate cause of the same. This is especially the case with the wisdom teeth. A young lady has a sweet voice and is considered a charming musician; after a time she becomes teazed with sore throat; it comes and goes in a most tiresome manner; gradually the throat becomes rough, the tonsils enlarge, and the character of the voice is spoiled; and the more you do to the throat, usually the worse it becomes. Caustic silver is applied, then iodine, and a score of other applications. These are all useless, if the wisdom teeth are covered with a thick, red, and at times tender gum, for here is the fault: so long as these teeth remain covered, or half-covered with gum, so long will your enlarged tonsils, thickened and roughened pharynx, and husky voice continue. Lance these gums freely, and if need be, cut away a portion from above the crown of the tooth; and after your tooth has come through, you will have no further difficulty with the throat or tonsils.

Another person will have a constant hacking cough—cough, cough, cough, all day long, relieved by nothing; or else it comes and goes in a most uncertain manner. The throat and tonsils are thickened, the teeth are not through, and you will say, there is no room for them to protrude. Should such be the case, you had better extract the second molar, if it is decayed, for then the third will most likely take its place. If it is not, it will soon become so, provided there be really no room for the wise tooth to get out, by reason of the pressure exerted upon it. If you are assured, by the continuance of severe pain in the wisdom tooth (or from some other cause,) of its decay, you had better extract it at once, if practicable, and by so doing you will relieve your patient from a large amount of pain and inconvenience.

In some cases the wisdom teeth are cut with convulsions, with severe cephalalgia, with different forms of neuralgia, with long-continued vomiting, with diarrhea, or several other symptoms of disease, which usually continue, more or less, until the teeth are through the gum, and the latter has receded so that it is free from pressure.

Abscesses near the angles of the jaw occur from this cause, earache, and abscess within or behind the ear, opthalmia in various forms, and sore throat, the latter most likely some time during dentition, in every case.

I have been led to pay considerable attention to the disorders which accompany dentition of the wisdom teeth, from the great frequency with which I have met with sore throat and enlarged tonsils when this process has been going on. As assistant surgeon to the large military seminary of the Honorable East India Company at Addiscombe, I have had great opportunities of observing this process among the gentlemen cadets at that establishment; and I have every reason to believe that a great number of the cases of sore throat, frequent headaches, continual coughs, and several other troublesome disorders, as opthalmia, discharge from ears and nose, have had a connection with irritation arising from dentition of the wisdom teeth.

Before leaving this part of my subject, I will say a few words regarding cancrum oris, or cankers, as it is commonly called. It commences by ulceration inside the cheek, quickly followed by gangrene, and accompanied by a copious secretion of fetid saliva; it is caused by inflammation of a low character, and is a consequence rather of debility than active inflammation. It rapidly sloughs through the cheek until it opens upon the outer side. The gums and alveoli are seriously involved, the teeth become loose and carious, and drop out; abscesses form in various parts, and make openings for themselves in different directions; portions of the maxillary bones die, and if the patient recovers, a long and tedious process has to be gone through to allow of the exfoliation of the dead portions of the bones. This disease is, happily, not a frequent one in this country; it is best met by free living, and invigorating remedies—wine and quinine, &c., whilst fresh air will, in importance, be second to none, except highly nutritious food.

The next subject which will engage our attention, is caries and its allied disease—necrosis. You will recollect I told you, in my first lecture, that caries represented, or was analogous to ulceration in soft tissues, and necrosis represented mortification. The same distinction will apply as well to the teeth as to the other bony structures of the body. There will, however, be some little difference in the actual progress of caries in a tooth, as compared with caries of a vertebra or other bone, having a cancellous structure, for, I may tell you, that true caries is much more usually found in soft and cancellated bones, whilst necrosis is more usual in the hard bones: thus, necrosis is common in diseases of the maxillary bone, but caries is rare. I believe, Mr. Tomes is our best authority upon the causes of caries, and I cannot do better than introduce you to his opinions upon the subject as detailed in his valuable work upon the teeth, which I should strongly advise you all to study with diligence and attention, although I differ a little with him in some of his points.

Mr. Tomes defines caries to be "death and subsequent progressive decomposition of a part or the whole of the tooth;" and according to his theory, two circumstances are necessary for complete dental gangrene or decay; viz. "a concurrence of dead dental tissue, and a condition of the oral fluids capable of decomposing the dead part." The vitality of the dentine being the groundwork of Mr. Tomes' theory.

He says that caries is attended with a vital physical change in the dentine itself; the dead is not separated from the living part of the tooth, but it is circumscribed by the consolidation of the living that is next to the dead. The tubes become filled up; they are rendered solid, and the circulation is cut off from the dead mass by the obliteration of the tubes.

Mr. Tomes also states, that if a carious tooth be examined with an eye-glass, the carious portion will be seen to be circumscribed by a translucent line, which is caused by the consolidation of the dentinal tubes, and he views this change as analogous to that which occurs in soft parts when affected with inflammation leading to abscess.

This opinion, to my mind, is opposed to his definition, and will correspond rather to the ulcerative process than complete death of the part; so that merely differing in words, we quite agree in description.

Mr. Tomes also gives as another proof of the vitality of the dentine, "the fact of irritation being transmitted in cases of caries to the pulp, whereby it is induced to renew its formative functions, and recede from the source of annoyance behind a barrier formed at the expense of its own substance."

According to his view, the first process of caries consists in the death of a portion of the tooth; the second, in the decomposition of the dental tissues. "The dentine," he says, "from abnormal action, loses its vitality, and with the loss of vitality the power of resisting chemical action; and the dead part, therefore, under favoring circumstances, is decomposed by the fluids of the mouth. The physical changes which mark the progress of the disease, are first observable under the enamel; which latter, over the affected spot, is either slightly discolored, or unusually opaque and white: the diseased dentine becomes brown, more or less marked, according to the rapidity with which the decay has advanced; the slower the process the deeper will be the color of the carious portion, and *vice versa*. The decomposed condition advances gradually from the surface towards the centre, invariably taking the course of the dental tubes; and as the various branches of these converge in their passage inwards to join the main tubes, the diseased mass is necessarily of a conical form. After a while, the surface of the dentine immediately under the enamel becomes softened by the loss of its earthy ingredients, the enamel itself affected, and no longer supported by the subjacent dentine, falls in, and a cavity is formed in the tooth which increases in depth, following the course of the tubes, and likewise extending laterally. By a continuance of this destructive process, an opening is formed in the pulp cavity, and eventually the whole of the crown of the tooth is destroyed.

"Sometimes, at this stage of the process the disease becomes arrested, there is more vitality in the fangs, they resist the ulcerative process, and are proof against the influence that destroyed the crown. In these cases, the inside of the cavity thus formed, is found of a dark brown color, bright and hard, like ivory or eburnated bone, and by this means the fangs are retained, their vitality being preserved, and the second variety of diseased bone, viz. necrosis, is not perfected. Should, however, the whole of the pulp be destroyed—should the ulcerated process remove or render useless the dental nerve, then necrosis of the whole tooth results, and eventually the whole must be got rid of, or it will be a thorn in the maxillary jaw, a source of constant suffering and uneasiness to the unfortunate owner."

I would here say a few words upon the custom of destroying the nerve before proceeding to stop the tooth. It is a mistake to suppose the nerve destroyed, for if it were so it would be useless to stop the tooth: a few weeks at most will lead to the death of the tooth, and the owner will be ready to get rid of it, and blame (deservedly) the dental surgeon who had, as he supposed, badly stopped his tooth. I should therefore strongly advise you to study these processes, freely examine into every point connected with the physiology of the teeth and their microscopic structure; consider well the pathology of ulceration and gangrene; bring all the general facts of inflammation to bear upon these processes, and you will then be in a proper and safe condition to study the local effects of disease in the dentine and *crusta petrosa* of the teeth themselves. It is from dental physiologists neglecting the study of general pathology that so much error—so much seeming contradiction has arisen as to the causes and progress of dental caries. Of course, if the nerve is destroyed, or if the first inflammation is sufficiently intense, the whole of the tooth becomes implicated; it is necrosed, and all your efforts will be useless to preserve it in the mouth, and stopping such a tooth would be unpardonable ignorance. You may, by constant cleanliness, by attention to the secretions of the mouth, by occasional applications of mild astringents, you may, I say, stay the progress of caries, and assist in producing the stage of eburnation mentioned by Mr. Tomes; but no aid can restore dead bone. You may heal an ulcer on the leg, but you cannot restore vitality to a mortified part; ulceration will be set up around it in the same way that ulceration arises in the gum around the crown of the tooth, preparatory to the attempt on the part of nature to get rid of useless and offending matter.

I would also tell you, you should study the opinion of Mr. Donaldson Mackenzie; there is a paper by him upon the predisposing causes of dental caries, published in the Medical Times and Gazette, October 28, 1854, which contains some useful remarks, even if not perfectly correct. His opinion is, that caries always commences on, and not in the teeth, and founded upon this opinion is his advice to use the file at an early period of the disease. You may study his paper, but afterwards judge for yourselves.

I will, before quitting the subject of caries of the tooth, give you the opinion of another author upon the pathology of caries, that of Mr. S. J. Salter, as detailed in a paper read before the Medico-Chirurgical Society, June 26th, 1855, which is well worthy your perusal.

Mr. S. J. Salter states, "When dentine becomes carious, the pulp becomes impregnated with calcareous matter, which produces a change in its physical properties, it first becomes firmer than natural, then hard and elastic, then perfectly hard and brittle according to the degrees of calcification which occurs intrinsically in the substance of the pulp, and not on its surface, differing in this respect from primary, and the other two forms of secondary dentine, it is displayed at sundry isolated points which are not interstitial deposits of earthy matter, but consists of animal matter impregnated with earth. As the calcification advances, the points enlarge, and at length form a dense coherent mass, which is *osteo dentine.*" "When the pulp has become perfectly calcified, the functions of the nerves have ceased, and the structure is without sensation; but at times, instead of osteo dentine, the pulp is converted into crusta petrosa, the process, though morbid in its nature is reparative in its results." Caries of the maxillary bones is very rare compared with necrosis; I have never met with a case; but of the latter disease, instances are very frequent. I can recollect one or two instances in the days of my pupilage, when I used the key for the purposes of extraction, fracturing a portion of the alveolar processes so irretrievably, that it was afterwards removed, necrosed; and I feel assured that the same mishap must have happened to every heavy-fisted apprentice, whose duty it is to draw teeth without instruction, and to judge as to the proper treatment of toothache, as if the knowledge came into his head as naturally as the knowledge of good and evil, and without regard to the future effect upon the poor unfortunates who happened to fall in the way of his tender mercies.

Thank goodness, however, that day is drawing to a close, the sun of wholesale tooth-drawing is setting; and upon you, gentlemen, and those honorable men associated with you as the promoters of this Dental College, will devolve the duty of responding to the public call now being made for educated dentists.

Necrosis of the jaw may arise then from injury, or from the violence of inflammation; the injury may be so severe as to destroy the chance of recovery, although the jaw may be very severely fractured, and yet necrosis will not follow, especially if the health is in a good state, for it is a condition more depending upon the general health than altogether upon local causes. In speaking of abscess, I alluded somewhat to the small sequestra of bone which sometimes follow that process. It may, however, involve a large part of the jaw-bone, more especially the lower jaw; matter continues to form around the dead part, and sinuses form in the cheek and beneath the ear, leading to a very disgusting state of things, which will cause the patient to submit to any thing to get rid of the offender. The probe passed into these sinuses, impinges upon the dead bone, and when this has been discovered, no time should be lost in removing it, being careful to be assured that it is dead bone, and not, as sometimes happens, a dead tooth or teeth. Those of you who may happen to be engaged in any cases requiring such operations, may consult Mr. O'Shaughnessy's work on Diseases of the Jaws, with advantage. He goes into the details of the operation for removal, and gives several very interesting cases.

I ought here also to mention the effect of phosphorus upon the maxillæ, causing a disease which has made its appearance at our hospitals within the last few years, and is restricted in a great measure to those persons employed in lucifer-match factories. The disease only attacks persons having carious teeth, the phosphoric vapor being enabled to enter into direct contact with the periosteum, on which its irritating influence is subsequently exerted. The disease consists essentially of inflammation of the periosteum, terminating in necrosis, or death of the bone. A low periosteal hypertrophy, says Mr. Simon, who has described this disease, with gradual alteration of tissue, and a laminated growth of bone in the periosteum, and beneath it, constitute the first stage of the disease.

This stage is not attended with much suffering, and usually the patient considers himself tormented with toothache; presently an attack of acute inflammation arises, great tumefaction and severe pain come on, the soft parts yield, the jaw, more or less completely, is at once struck with necrosis, and is seen lying in the midst of disorganized tissues, which pour out a profuse and insufferably-fetid discharge. The case is now one of necrosed bone; the separation takes place in the usual way, and if the patient survives the constitutional disturbance of the second stage, the knife of the surgeon will be necessary to set free the dead bone. Now, in cases of this kind, prevention is better than cure, and whenever you have patients who are engaged among phosphorus fumes, be careful to warn them of the danger of inhaling those fumes, if they have carious teeth. If their dental organs are sound, there is no fear from this source. Should the disease have commenced, free incisions must be made along the line of the gums, down to the maxillary bones themselves; free incisions; and the remaining treatment must be managed upon general principles.

The next subject that will engage our attention is neuralgia; you will all know what I mean by neuralgia, without any close definition of the term; in fact, I do not know a physiological definition, and it is not in itself a precise term, but it will very well serve our purpose. It is not known in what part of a nerve the pain has its seat, or in what manner the nerves terminate, so that I cannot even fix the precise seat of the pain which constitutes neuralgia. It is an acute pain, commencing suddenly, occupying a certain spot, and radiating by pangs, or flashes, to the surrounding nerves, and in its radiations it follows the threads of the nerves, without extending to the neighboring structures. There is no heat, no redness, no swelling, and the pain, after a continuance of a longer or shorter time, suddenly abates, to return at some future period with similar, or even greater intensity. The cause of neuralgia, like its seat, is unknown, at least the exciting cause, for in some cases the predisposing cause is more manifest. There are two classes of cases, one intermittent and the other pure, the latter being independent of every cause. It usually occurs in pale, luco-phlegmatic persons, and seems to be a disease of debility. It is therefore very common among those suffering from exhausting causes, such as over lactation. Some ladies will suffer from most violent pain after every time the child takes the breast, the pain evidently having its origin in debility, the result of the drain from the breast; but it may not acknowledge any such cause, and will sometimes be brought on by such slight touches or movements, as warrant the assumption of some magnetic condition giving rise to it.

The intermittent neuralgia is frequently a symptom of disease, and is often allied to ague, having its origin in similar causes, such as marsh miasma, and other sources of malaria; it usually comes on at a stated period, with great regularity, and seems to take the place of a regular ague fit.

Now neuralgia of both kinds may be seated anywhere. It will be frequently present in some portion of the dental nerve, as well as any other branch of the fifth pair. You will be frequently called upon to remove teeth which are the subject of this disease, and if you do not make out clearly the nature of the disease, and if you accede to his (your patient's) wish, and draw the tooth indicated by him, he will probably call upon you on the following day, and say you have drawn the wrong tooth.

You will not usually find the diagnosis difficult; except the pain, there is an absence of all the symptoms of inflammation. The pain comes suddenly, and goes as quickly. The character of the pain will guide you: you will know it from toothache, the result of exposed or inflamed pulp, by the pain in the latter case being centralized in one tooth, which is always tender when it is struck; and there is always a hole in it, and if the exposed pulp be touched, it is found to be exquisitely sensitive. Whilst if the case is one of pure neuralgia, none of the teeth will be tender when touched, and the patient will be enabled to eat, even without increasing the pain, unless indeed the neuralgia depend upon a local cause, peculiar to the teeth themselves, or, as is occasionally the case, from the presence of a foreign body impacted into the jaw, and irritating a nerve, but not exciting sufficient inflammation to produce its own expulsion. When the neuralgic affection depends upon some local irritation of the dental nerves, or from pressure upon a branch of that organ, it generally has its origin in a condition of the fang called exostosis. This is a thickening of the periosteum of the fang by the deposit of true bone, or at least substance analogous to the *crusta petrosa,* so that the fangs of the teeth affected with this condition, become thicker and larger at their apices, than in the middle of their length, and, as you may suppose, cannot be drawn very easily from their beds; an attempt, therefore, to extract will frequently end in failure, the extremities of the fangs are left in their sockets, and the neuralgic affection remains unrelieved. If you have diagnosed your case correctly, great care in extraction may be successful, in which case the neuralgia departs, not again to return.

Should you be unsuccessful, I do not know of any thing more efficacious than large doses of iodide of potassium, given until the system is fully under the influence of the iodine. I believe I have cured some cases of neuralgia, dependent upon exostosis of the fangs, by this medicine continued for some time.

If you believe that your case is caused by some exhausting drain, as over lactation, you must advise your patient to wean her child, and take abundance of good diet. Quinine and steel will also be useful in various forms and combinations, the citrate of iron and quinine, when other things do not forbid its use, will be especially beneficial. When the neuralgia depends upon malaria for its origin, when it is intermittent in its character, you will generally cure it by iron and quinine, with port wine, taking care to see that these medicines do not interfere with the action of the liver and bowels.

If these remedies do not suffice, you had better recommend change of air, as some local cause of malaria may interfere with the action of your remedies. But at times you will meet with cases which will baffle all your skill, will not yield to any of your remedies, you may divide the nerve, (which has been repeatedly done,) the affected branch of the nerve, without relieving your patient in the least; and what to do then I really cannot tell you, but as necessity is the mother of invention, I hope the day is not far distant when some remedy may be discovered for these obscure affections of the nervous system, which at present are the *opprobia medicorum.*

You will understand from what I have said, that nine-tenths of the cases called facial neuralgia are not really so; they have their origin in inflammation of some portion of the nerve pulp, or the dental structures, and are not cases of true neuralgia. It will be important to make this distinction, as the remedies that are correct for neuralgia proper, will fail if your case depends upon some other state.

Sometimes a severe neuralgic affection is caused by the condition of the secretions of the mouth. You will recollect I told you that Mr. Donaldson Mackenzie believes that caries always commences on and not in the teeth. Whether the term always is correct is a matter of opinion at present; I think he is not correct, but undoubtedly it is so sometimes. Undoubtedly the enamel may be removed from the surface of the dentine, and then the dentine will become carious, just as the end of the finger would ulcerate if the skin was taken off, and in the end your tooth will be destroyed. Now it occasionally happens that we meet with persons in whom the enamel is almost all removed, and if we test the secretions of the mouth in these persons we shall find them to have at times a strong acid reaction, and this will be especially the case if they are suffering from neuralgic pains; it seems as if the acid secretions of the mouth were gradually destroying the teeth, exposing the dental pulp, and exciting neuralgic pains from the mere contact of irritating acid secretions, for we frequently find the pains relieved by washing the mouth with an alkaline lotion.

It is by forming an acid (the lactic acid) that sugar acts injuriously upon the teeth, and those persons who are very fond of sweets, will be sure to suffer unless they are careful to remove all trace of sugar from the mouth, for the warmth of the position soon excites a fermentative action, and lactic acid is developed. In the same way also many persons suffer severely from diseased teeth after great illness, as for instance after fever. During the existence of typhus fever the secretions of the mouth are acid, and these act so far upon the enamel as to make minute holes through it, which become carious when the patient has recovered and the teeth have been freely used. In the majority of these cases the state of the teeth will be referred to the medicine that has been given, and it is a very common custom for dentists to say as much to those who consult them. I have frequently met with persons who have been highly indignant with their medical attendants for spoiling their teeth. They might just as well blame them for the loss of their hair, which also came off, or the scaling from their skin; scarcely ever have I met with an instance in which I could only say the injury to these teeth has been caused by medicine, and the only possible cases in which such a charge could be substantiated would be such cases have been profusely salivated, and also those in which the strong mineral acids have been prescribed without giving due precautions in regard to the method of taking them.

In the first instance, viz. that of profuse salivation, we shall be most likely unable to judge how far the action of the mercury was the cause of the patient's appearance before you, inasmuch as the action of mercury is so well understood now, that it is never pushed to that extent, unless it is absolutely necessary to save the life of the patient. It will be wise, therefore, for you to represent as much, and not to be the means of continuing a false impression on the mind of your client.

I would recommend you here, again, to be especially cautious in your behavior towards the members of the medical profession; never advance an opinion that may perchance be untenable, if it reflects at all upon the conduct of the medical attendant of your patient, or the treatment he has pursued; you may rest assured that it will be repeated again, with embellishments and variations, and will produce ill will towards yourselves individually, and if the medical man is once obliged to act as dentist to his poorer patients, it will undoubtedly react injuriously against the interests of the dental profession. You must recollect that you will be—you must, by the very force of circumstances—regarded as interlopers by the men of the old school, and also by the greater number of my professional brethren whose patients cannot generally afford to pay the charges of a first-class dentist. A few years, however, will, I feel assured, see a change come over this part of our story, provided you carry out the intentions of the founders of this college, and make your profession what it ought to be, viz. recognized as a learned one; then will there be no fear of your being regarded in any other light than as useful, and I may say, powerful auxiliaries to the medical profession.

[*Lond. Quar. Jour. Dent. Sci.*

**(To be concluded in our next.)**

